# Serum creatinine in predicting mortality after paraquat poisoning: A systematic review and meta-analysis

**DOI:** 10.1371/journal.pone.0281897

**Published:** 2023-02-22

**Authors:** Wei Huang, Zheng Zhang, Yuan-Qiang Lu

**Affiliations:** 1 Department of Emergency Medicine, The First Affiliated Hospital, School of Medicine, Zhejiang University, Hangzhou, Zhejiang, People’s Republic of China; 2 Key Laboratory for Diagnosis and Treatment of Aging and Physic-chemical Injury Diseases of Zhejiang Province, Hangzhou, Zhejiang, People’s Republic of China; Maulana Azad Medical College, INDIA

## Abstract

Although the prognostic value of blood creatinine levels in patients with paraquat (PQ) poisoning has been studied for a long time, the results are still controversial. Therefore, we performed the first meta-analysis to comprehensively assess the value of blood creatinine in predicting the prognosis of patients with PQ poisoning. We searched PubMed, EMBase, Web of Science, ScienceDirect, Cochrane Library, China National Knowledge Infrastructure, China Science and Technology Journal Database, and China Online Journals to identify all relevant papers published up to June 2022. Data were extracted for pooled analysis, heterogeneity testing, sensitivity analysis, publication bias analysis, and subgroup analysis. Ultimately, 10 studies involving 862 patients were included. The I2 of diagnostic odds ratio (DOR), sensitivity, specificity, positive likelihood ratio, and negative likelihood ratio of this study were all greater than 50%, which showed the existence of heterogeneity in this study, and a random effects model was used for the combination of the above five effect sizes. Pooled analysis showed a high predictive value of blood creatinine for prognosis of PQ poisoning [pooled DOR:22.92, 95% confidence interval (CI):15.62–33.65, P < 0.001]. The combined sensitivity, specificity, positive likelihood ratio, and negative likelihood ratio were 86% (95% CI: 0.79–0.91), 78% (95% CI: 0.69–0.86), 4.01 (95% CI: 2.81–5.71), and 0.17 (95% CI: 0.12–0.25), respectively. Deeks publication bias test revealed there was publication bias. Sensitivity analysis showed no significant differences in the estimates of impact. Serum creatinine is an effective predictor of mortality in patients with PQ poisoning.

## Introduction

Paraquat (PQ), an organic heterocyclic pesticide, is highly toxic to humans [1, 2]. Many people ingest PQ as a suicide agent, since PQ is readily available and inexpensive in most countries and has a very high mortality rate after poisoning, especially in Asian countries where PQ poisoning is a serious public health problem [3, 4]. PQ poisoning can lead to multi-organ failure, with the main organs damaged being the kidneys, liver, and lungs [5], but the mechanism of poisoning has not been fully determined. The main treatment modalities for PQ poisoning are in vitro removal, immunosuppression, and antioxidants, and although many treatments have been developed, the efficacy of these treatments is uncertain [6, 7]. Because of the extremely high mortality rate from PQ poisoning, researchers have developed a number of survival predictors.

Some studies have shown that plasma PQ concentration and severity index of PQ poisoning (SIPP) can be used as reliable predictors of mortality from PQ poisoning [2, 7], but plasma PQ concentration is expensive and difficult to detect. Some studies have shown that sequential organ failure assessment (SOFA) score and acute physiology and chronic health evaluation II (APACHE II) score can be used as prognostic indicators of PQ poisoning [6–11], but they are comprehensive indicators that have challenges in calculation as well as limited usability and reliability in the early stages of poisoning, and are hardly representative of the patient’s early condition. In addition, the toxic dose can be used as a prognostic indicator [12], but it is unable to evaluate an accurate value. Thus, there is a need for a better prognostic indicator that is simple and accurately reflects the true condition of the body. The kidney is the main organ for excreting PQ, and the early stage of PQ poisoning can lead to kidney damage [13, 14]. Meanwhile, the blood creatinine level of the organism reflects the renal function [15]. Several studies have concluded that blood creatinine levels can be used as a prognostic indicator of PQ intoxication. However, the sample size of a single study was small and the statistical efficacy was low, we used meta-analysis to improve the statistical efficacy and to investigate the predictive value of blood creatinine on mortality from PQ poisoning in a comprehensive manner.

## Methods

As a meta-analysis, this study followed the Preferred Reporting Items for Systematic Reviews and Meta-Analyses (PRISMA) checklist. Since we only review previously published research, there are no ethical issues involved in our study. In June 2022, the study protocol was registered in the International Prospective Systematic Review under protocol number: CRD42022335006.

### Search strategy

The following 8 databases were used for this study: PubMed, EMBase, Web of Science, ScienceDirect, Cochrane Library, China National Knowledge Infrastructure, China Science and Technology Journal Database, and China Online Journals. The literature prior to 2022.6.1 was searched using the terms PQ and creatinine; see the Appendix for specific search strategies (S1 Table).

### Inclusion and exclusion criteria

The inclusion criteria were as follows: studies covering serum creatinine in patients with PQ poisoning in both the survival and death groups; patients of any age diagnosed with oral paraquat poisoning (although inhalation or contact with the skin were also routes of poisoning, these were not the focus of the study); studies focused on people with moderate to severe poisoning, as typically in clinical practice, mild paraquat poisoning (which tends to resolve on its own) and extreme severe poisoning patients often did not receive treatment; studies with sample sizes ≥ 30 cases; studies covering mortality within 90 days of paraquat poisoning; data related to serum creatinine could be calculated as true positive, false positive, true negative, and false negative. The exclusion criteria were as follows: reviews, commentaries, conferences, letters, editorial abstracts, meta-analyses, animal studies, informal publications, and case reports; literature with insufficient data; literature with implications for the stability of the combined results. In case of duplicate data publication, studies with the most recent year or larger sample size were selected.

### Data extraction

The required content was extracted independently by two researchers, and the extracted content included: first author, year of publication, cut-off value, blood creatinine level, sample size, number of survivors and deaths, cut-off value, sensitivity and specificity of blood creatinine to predict PQ mortality and study type. Discrepancies were resolved through third-party coordination.

### Risk of bias

We used quality assessment of diagnostic accuracy study 2 tool (QUADAS-2) separately to assess the quality of this study, and the risk of bias was graded as low, unclear, or high, with any inconsistencies resolved through discussion.

### Statistical analysis

Commonly used software for diagnostic accuracy meta-analysis include Stata, R, and meta disc. The packages that can do diagnostic accuracy test meta-analysis in R are "meta4diag, bamdit, hierarchical summary receiver operating characteristic (HSROC), metamisc, mada, CopulaREMADA, and Metatron", and the first three packages are based on Bayesian theory. The effect sizes in meta-analysis of combined diagnostic accuracy tests with these R packages above have certain drawbacks, for example, the meta4diag package cannot perform heterogeneity tests, the HSROC package cannot draw forest plots, and although heterogeneity tests can be performed with the mada package, it cannot calculate the combined data. The study used stata17 for combining all effect sizes and sensitivity analysis, analyzed Deeks publication bias test and summary ROC (SROC) curves, also processed Review Manager 5.3 for quality assessment graphs. I2<50%, considered low heterogeneity, was used to combine effect sizes using a fixed-effects model; I2>50%, considered high heterogeneity, was used to combine effect sizes using a random-effects model.

## Results

### Literature search

**Fig 1:**
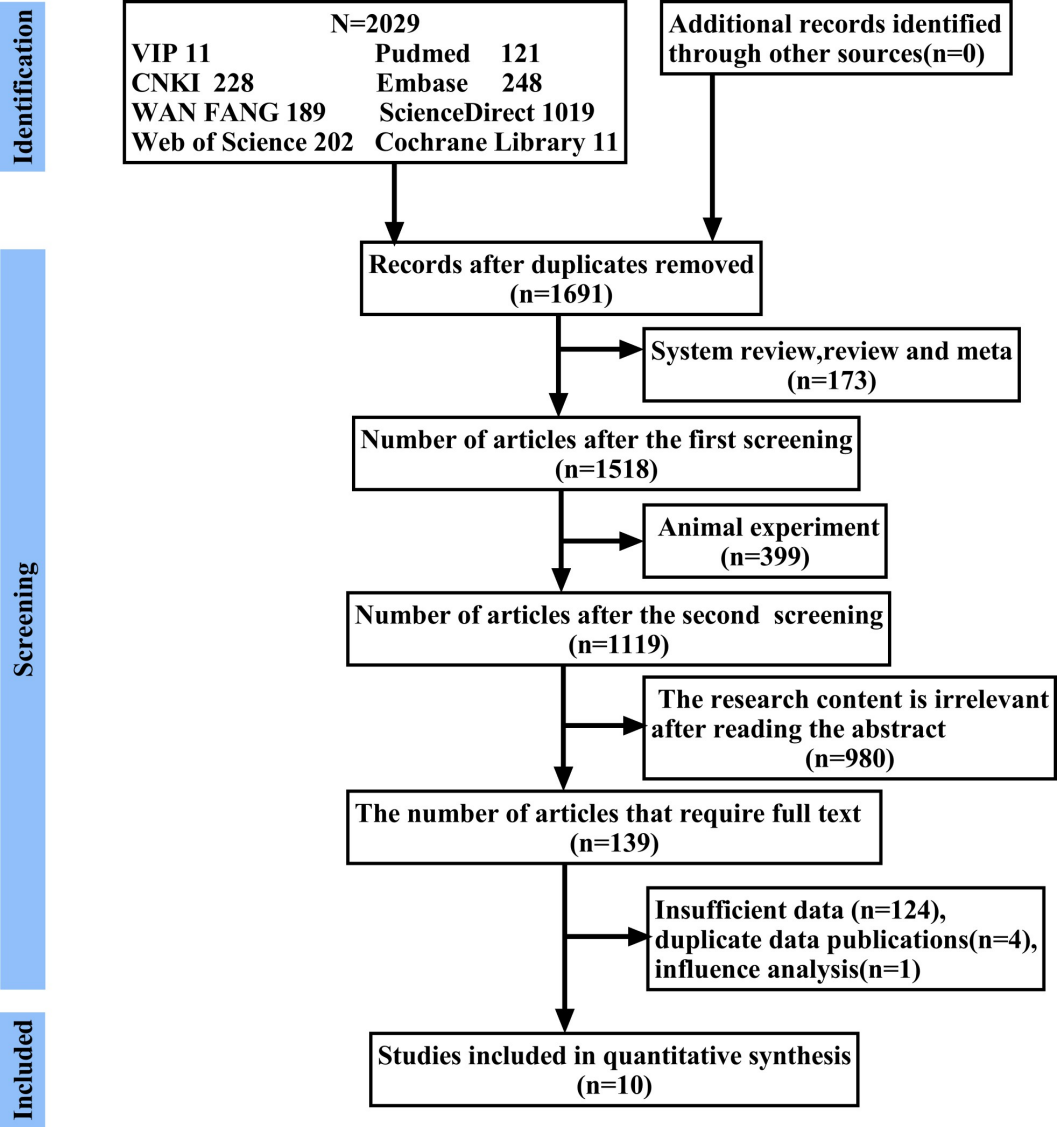
Flow chart of search process.

According to the search strategy, a total of 2,029 publications were found in PubMed, EMBase, Web of Science, ScienceDirect, Cochrane Library, China National Knowledge Infrastructure, China Science and Technology Journal Database, and China Online Journals. Duplicates, animal experiments, reviews, and meta-analyses were excluded successively, followed by reading the titles and abstracts to exclude irrelevant studies. Finally, after reading the full text, literature with duplicate publications, insufficient data, and influence on the stability of the results were excluded in turn, and 10 papers [16–25] with a total sample size of 862 were finally selected. The search process is shown in Fig 1.

### Characteristics of the included studies

The baseline characteristics are shown in Table 1. The 10 studies were published in the years 2011–2020, all retrospective, with a total sample size of 862 and a mean sample size of 86.2. Sample sizes ranged from 40 to 190 cases. Mortality rates ranged from 40.0% to 65.12%, with nine studies conducted in China and one in India. Five studies were published in English and the other five studies were published in Chinese.

**Table 1:** Characteristics of included studies.

First author	Publicatio-n year	Region	Study Design	Sample size	Morta-lity (%)	Cut-off (μmol/L)	Serum creatinine (μmol/L)		Study period
							**Survivor**	**Non-survivor**	
**Song**	2020	China	Retrospective	114	45.61	122	93.2 (73.0–124.2; 35.7–725.0)	277.0 (213.3–382.4; 67.8–1105.0)	2018–2020
**Zhao**	2020	China	Retrospective	108	59.26	74.5	62.50 (22.00)	104.00 (69.50)	2019–2020
**Wan**	2018	China	Retrospective	40	40	NA	NA	NA	2017–2018
**Su**	2020	China	Retrospective	190	62.63	102.1	82.71 + 42.55	88.94 + 112.72	2019–2020
**Chen**	2011	China	Retrospective	45	62.22	NA	54± 12	148± 24	2009–2011
**Xiao**	2017	China	Retrospective	58	43.10	110.7	64.03 ±23.52	149.34 ±86.03	2016–2017
**Wan**	2018	China	Retrospective	162	37.04	NA	75.06±50.81	130.54±126.16	2017–2018
**Wan**	2011	China	Retrospective	57	49.12	80.85	67.54±17.67	144.21±76.98	2010–2011
**Li**	2014	China	Retrospective	43	65.12	90.70	70.89±13.38	106.1±45.07	2013–2014
**Kavousi**	2017	India	Retrospective	104	43.27	172.38	NA	NA	2015–2017

NA: not available; Continuous variable is presented as mean ± standard deviation, median (interquartile range) or numbers.

### Quality assessment

**Fig 2:**
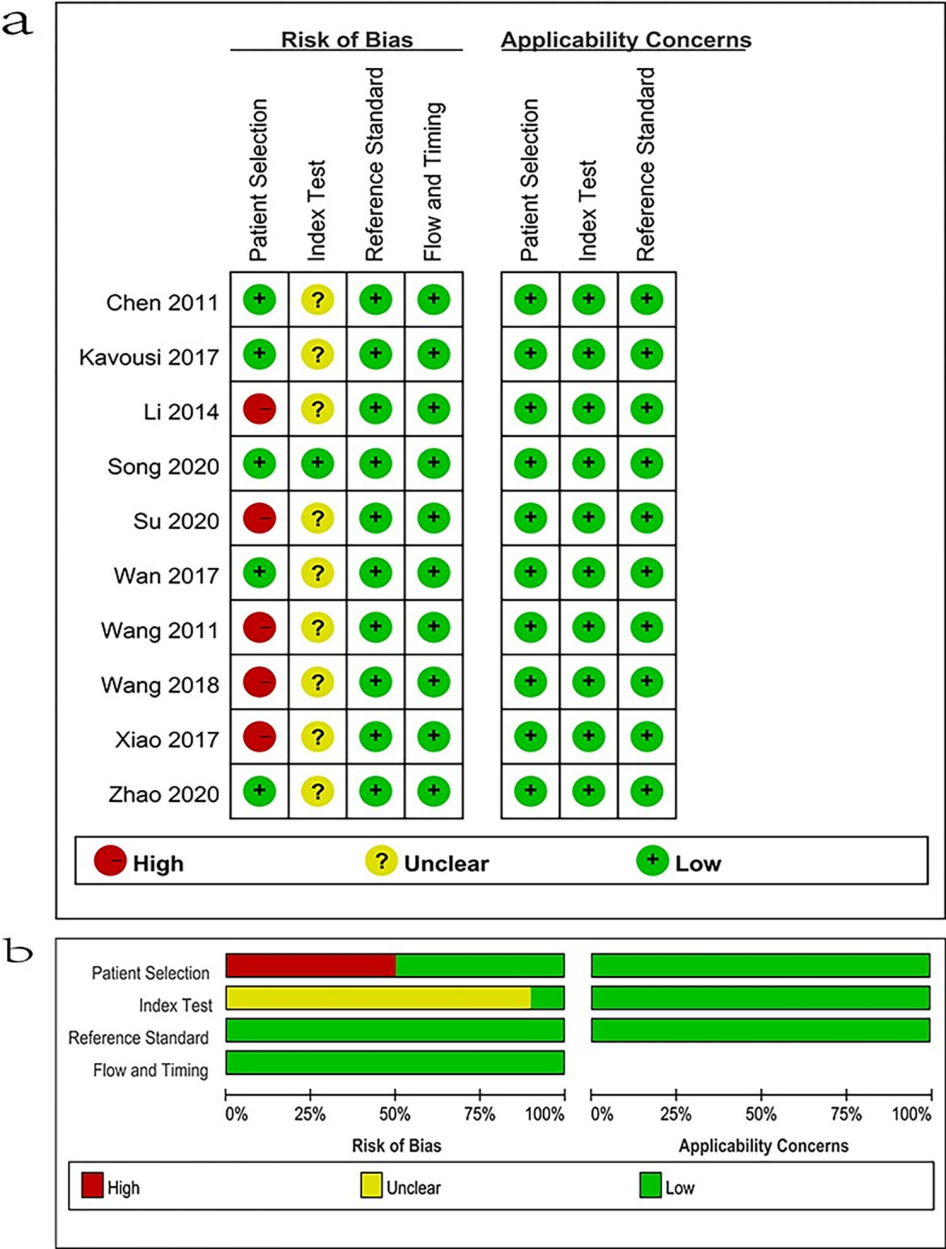
Risk of bias and applicability concerns. (+) denotes low risk, (−) denotes high risk.

The S2 Table presents the distribution of QUADAS-2 scores for the methodological quality of these 10 studies. All 10 studies showed moderate to high quality according to QUADAS-2 for quality assessment, so these studies are suitable for this meta-analysis (Fig 2).

### Meta-analysis of mortality

**Fig 3:**
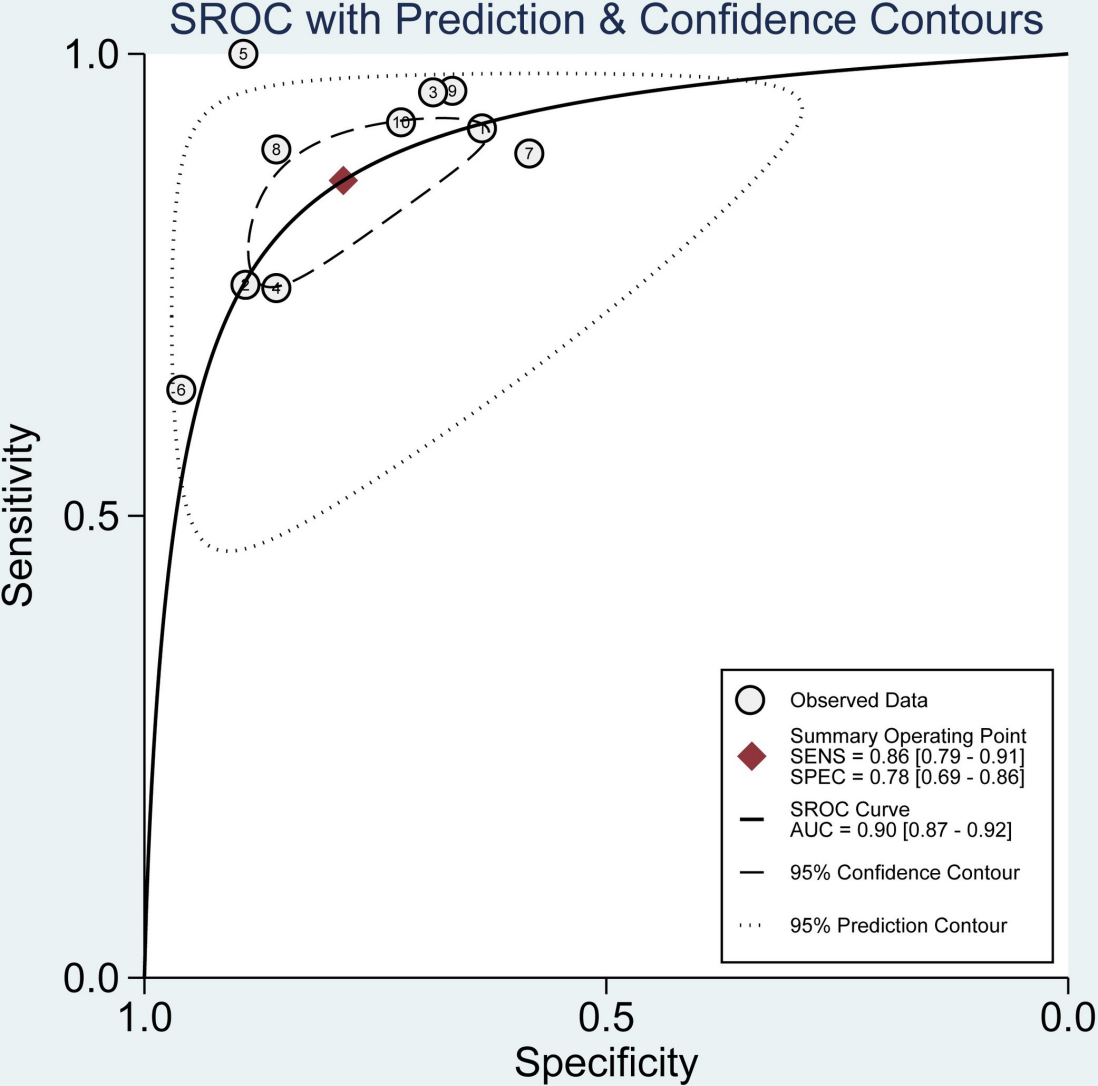
Summary ROC curve for the 6 included studies. Numbers in brackets are 95% CIs. ROC: receiver operating characteristic, AUC: area under curve, SENS: sensitivity, SPEC: specificity, CI: confidence interval.

The data were imported into meta disc1.4 software for analysis, and the spearman correlation coefficient between the log of sensitivity and the log of (1-specificity) was 0.365 (P = 0.30), which was not significant, implying that there was no heterogeneity due to threshold effect in this study. Furthermore, by plotting the SROC curves, there was no "shoulder arm shape", which further indicated that there was no threshold effect in this study (Fig 3).

**Fig 4:**
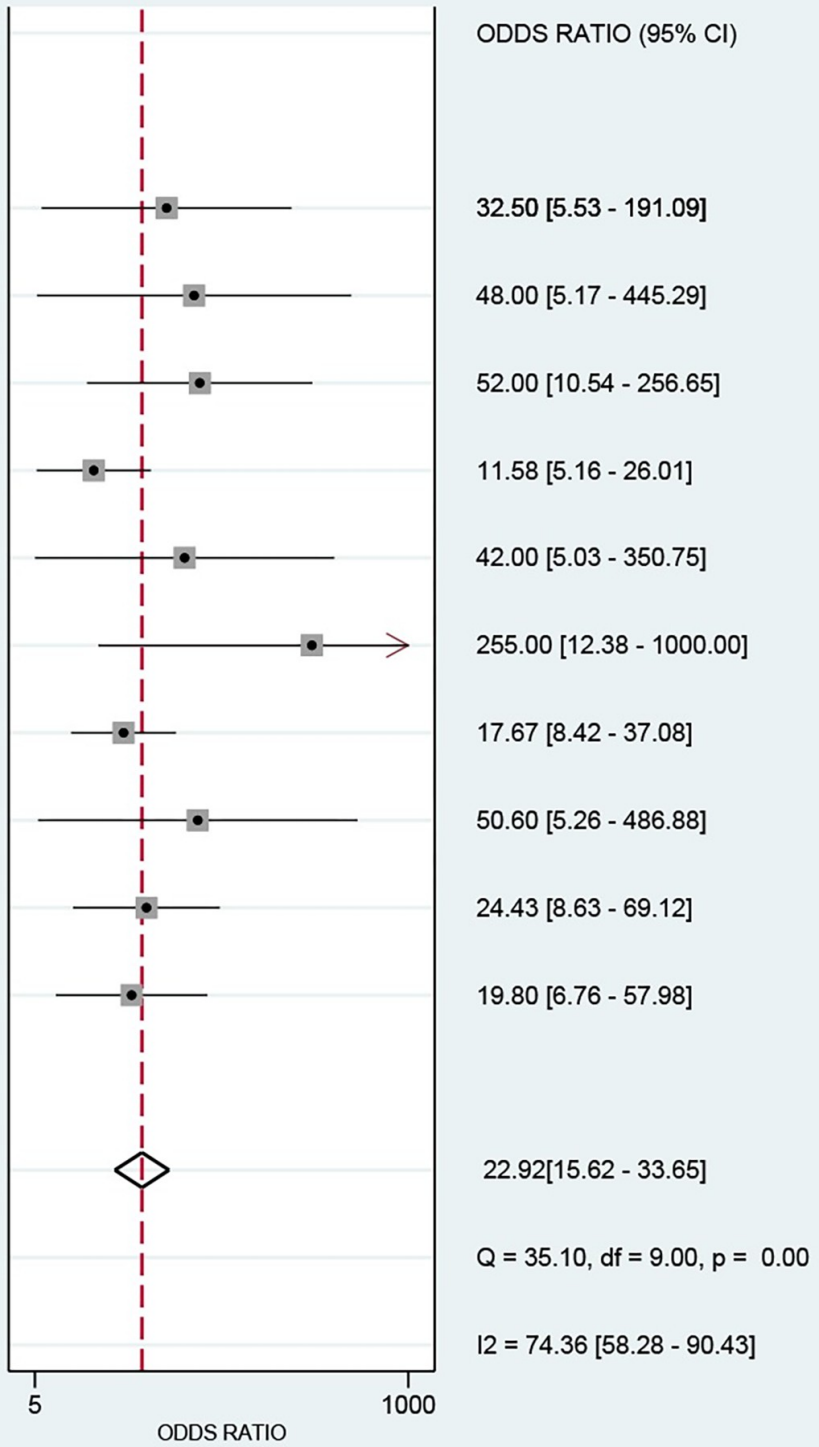
Forest plot for the association of serum creatinine and mortality of paraquat poisoning.

**Fig 5:**
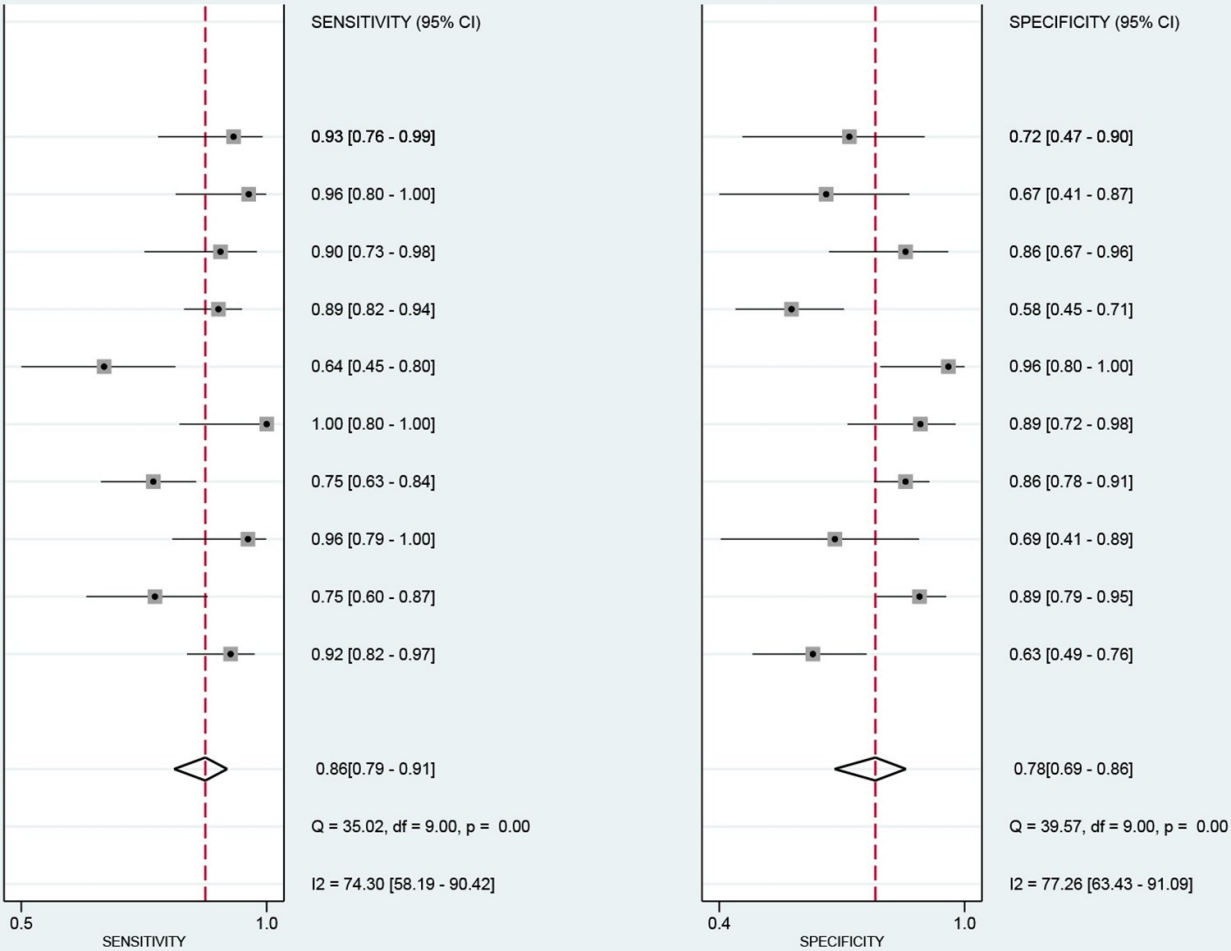
Forest plots of sensitivity and specificity of Serum creatinine in predicting mortality after paraquat poisoning. The diamond indicates the combined estimate from the included studies.

The data from this study were imported into stata17, and the Cochran-Q test for the diagnostic odds ratio (DOR) yielded Cochran-Q = 35.10, I^2^ = 74.36, P<0.01 (Fig 4), implying the existence of heterogeneity caused by non-threshold effects in this study. In addition, the I^2^ of sensitivity, specificity, positive likelihood ratio, and negative likelihood ratio of this study were all greater than 50%, which showed the existence of heterogeneity in this study, and the random effects model was used for the combination of the above five effect sizes. The combined sensitivity, specificity, positive likelihood ratio, negative likelihood ratio, and DOR were 86% (95% CI: 0.79–0.91; Fig 5), 78% (95% CI: 0.69–0.86; Fig 5), 4.01 (95% CI: 2.81–5.71; S1 Fig), 0.17 (95% CI: 0.12–0.25; S1 Fig), and 22.92 (95% CI: 15.62–33.65; Fig 4). The area under the curve (AUC) of the blood creatinine test was 0.90 (95% CI: 0.87–0.92; Fig 3), which meant that the diagnostic accuracy was relatively high.

### Heterogeneity, sensitivity analysis, and publication bias assessment

**Fig 6:**
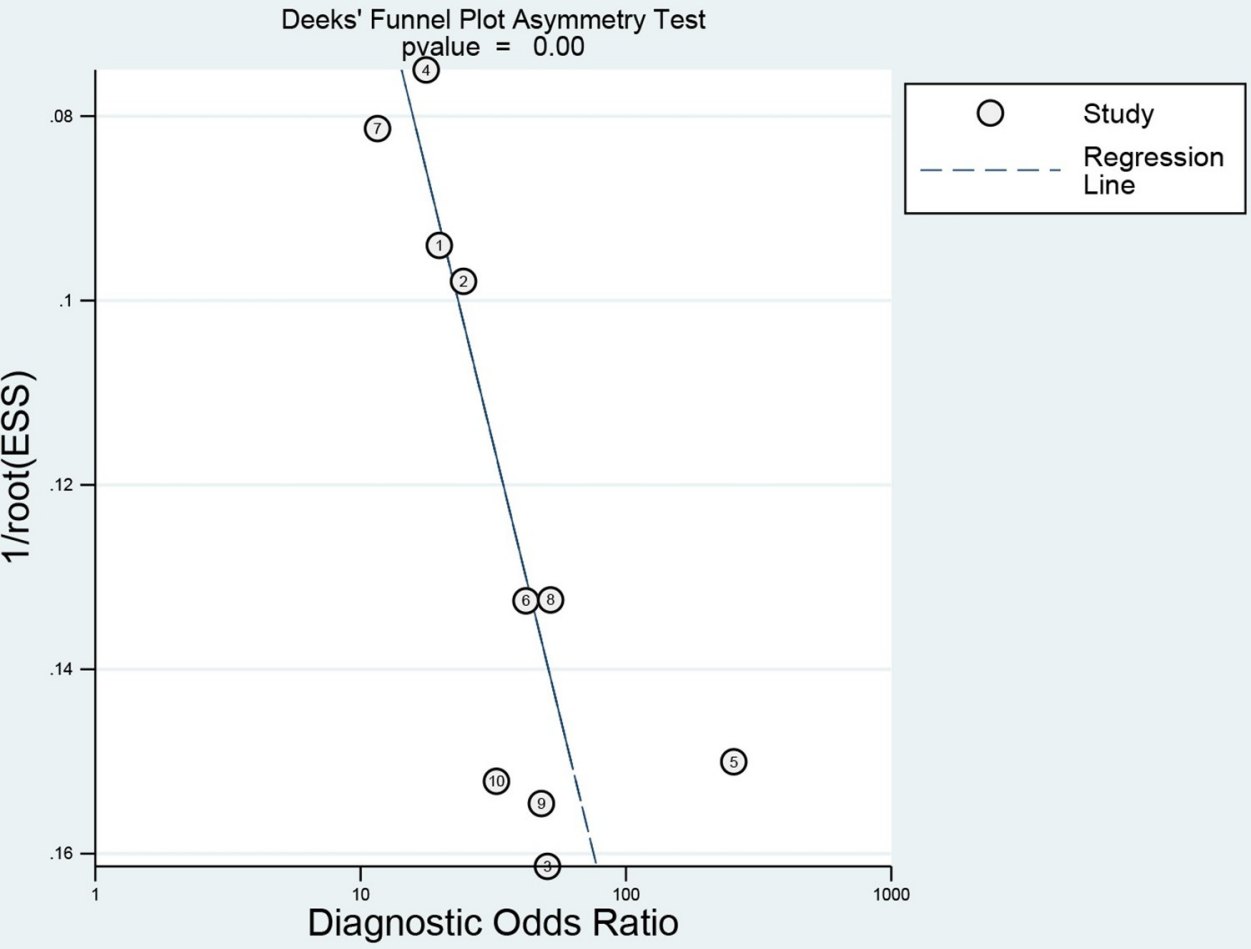
Deeks publication bias test.

**Fig 7:**
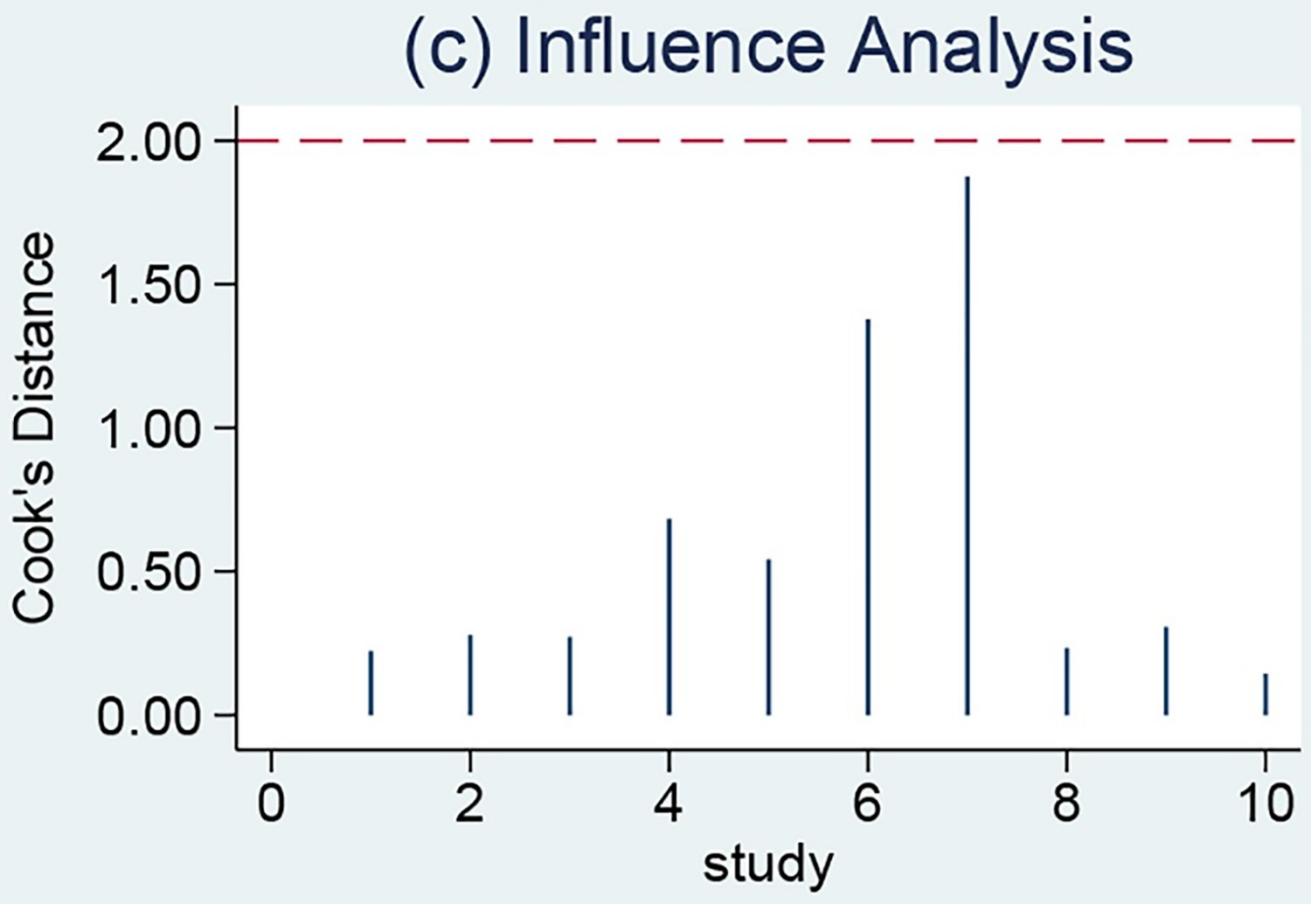
Sensitivity analysis of the relationship between serum creatinine and mortality of paraquat poisoning.

A meta-regression analysis was performed based on several covariates, including sample size, year of publication, percentage of men, mortality rate, and study area; however, the meta-regression results did not identify sources of heterogeneity (Table 2). P<0.001 in Fig 6 implied that the funnel plot was asymmetric and there was a publication bias in this study. In addition, one article with strong sensitivity was excluded by sensitivity analysis, and the remaining original study did not cause sensitivity in the arithmetic results (Fig 7). Taken together, the results of this study are relatively stable.

**Table 2:** Meta-regression analysis of potential sources of heterogeneity.

Heterogeneity factors	N	LRTChi2	Pvalue	I2	I2lo	I2hi
**Study Region**						
**India**	1	0.69	0.71	0	0	100
**China**	9					
**Mortality%**						
**≥50%**	4	1.30	0.52	0	0	100
**<50%**	6					
**Sample size**						
**≥100**	5	5.48	0.06	64	18	100
**<100**	5					
**Publication year**						
**>2017**	5	5.09	0.08	61	11	100
**≤2017**	5					
**Male%**						
**≥50%**	4	0.13	0.94	0	0	100
**<50%**	6					

### Translation to clinical practice

Fagan plots showed that when the pre-test probability of death from acute PQ poisoning by blood creatinine were 25%, 50%, and 75%, positive post-test probability were 57%, 80%, and 92%; and negative post-test probability was 6%, 15%, and 34%, respectively (S2 Fig).

## Discussion

This study aimed to find a simple and accurate prognostic indicator that reflected the true condition of the organism. This meta-analysis showed an association between blood creatinine levels and clinical prognosis in patients with PQ intoxication, which meant that the patient with PQ intoxication who had an elevated blood creatinine level had a significantly increased risk of death.

After PQ poisoning, it is mainly distributed in the lung, kidney, liver, muscle, and other tissues, while the absorbed PQ is mainly excreted through the kidney, and the renal PQ concentration is extremely high [26]. Reactive oxygen species produced by PQ poisoning cause the onset of cellular damage in the kidney proximal tubule, followed by lipid peroxidation, which disrupts membrane integrity and causes cell death [27]. This is a process that cells undergo swelling, degeneration and partial necrosis, interstitial congestion and edema, so that PQ poisoning often results in acute kidney injury, which in turn leads to renal failure, manifested by proteinuria and oliguria, and then progresses to acute tubular necrosis [28]. Direct oxidative damage to renal tubules by PQ can induce elevated blood creatinine [14]. The level of serum creatinine is mainly determined by glomerular filtration capacity, and a decrease in glomerular filtration capacity increases blood creatinine concentration, indicating that elevated blood creatinine levels are closely related to acute kidney injury and are a direct reflection of progressive kidney damage [29, 30]. As a result, blood creatinine concentrations can be monitored to detect renal failures and predict long-term outcomes [30–33]. From this study, the predictive value of blood creatinine for PQ poisoning mortality is high by DOR analysis. The PQ absorbed in the body is mainly excreted in its original form through the kidney, and if the kidney function is damaged at an early stage, it will greatly reduce the excretion rate of PQ, which will aggravate the accumulation and damage of PQ in lung tissues and organs, thus affecting the prognosis [34].

The sensitivity of blood creatinine to predict the prognosis of PQ poisoning was 0.86, which was higher than SOFA (sensitivity 0.72) [35], white blood cell count (sensitivity 0.75) [36], APACHE II scores (sensitivity 0.75) [8], arterial lactate (sensitivity 0.77) [37], base excess (sensitivity 0.78) [38], severity index (sensitivity of 0.84) [39], and plasma PQ concentration (sensitivity of 0.86) [39]. The AUC of the SROC curve was 0.90, indicating that blood creatinine had a high predictive value for the prognosis of PQ poisoning, and the results of the Fagan curve showed that when the pretest probability was 25%, the negative likelihood ratio was 0.17, and the posttest probability was 6%, indicating that the probability of having a certain disease was only 6%; when the pretest probability was 75%, the positive likelihood ratio was 4, and the posttest probability was 92%, indicating that the probability was 94%. It further confirmed that serum creatinine had a good predictive value for acute PQ poisoning.

There was significant heterogeneity in the accuracy assessment of this study, and meta-regression to explore some of the variables that might have contributed to the heterogeneity showed that none of these factors were the source of heterogeneity in this study. Therefore, the source of heterogeneity remained to be determined. In addition, there are some limitations of this study. First, the included literatures were retrospective and mostly case-control studies, which were susceptible to selection bias and information bias. Second, only 10 studies were included and some of them had small sample sizes. These might cause the limited generalizability and differences in race, region, disease stage, and treatment regimen across studies, which also led to bias. Third, there was a publication bias in this study, which might be related to the fact that most of the enrolled studies were conducted in China and the observational studies were more prone to publication bias. Finally, no unpublished studies were identified in our study, and no attempt was made to include articles in languages other than English.

In conclusion, this meta-analysis demonstrates that blood creatinine may be an independent prognostic indicator for patients with PQ poisoning. Therefore, clinicians should consider blood creatinine levels after PQ poisoning. However, a more adequate reason needs to be confirmed by clinical trials.

## Supporting information

S1 Table Search strategy. Recent queries in Databases on July 1 2022.
(PDF)

S2 Table Quality assessment. (PDF)

S3 Table Data extracted. (PDF)

S1 Fig Forest plots of positive likelihood ratio and negative likelihood ratio of serum creatinine in predicting mortality after paraquat poisoning. The diamond indicates the combined estimate from the included studies.
(PDF)

S2 Fig Analysis of the Fagan plot to evaluate the clinical efficacy utility of serum creatinine in predicting mortality. (A) Pre-test probability = 25%; (B) Pre-test probability = 50%; (C) Pre-test probability = 75%. Each Fagan plot contains a vertical axis on the left for the pre-test probability, an axis in the middle represents the likelihood ratio, and a vertical axis on the right represents the post-test probability. NLR: negative likelihood ratio, PLR: positive likelihood ratio.
(PDF)

S1 File PRISMA 2009 checklist. (PDF)

S2 File Raw data. (XLSX)
