# Supplementary material for: Serum creatinine in predicting mortality after paraquat poisoning: A systematic review and meta-analysis
Source: PLoS One. 2023 Feb 22;18(2):e0281897. doi: 10.1371/journal.pone.0281897 (PMC9946265; doi:10.1371/journal.pone.0281897)
Supplement: S1 Table — Recent queries in Databases on July 1 2022. (PDF) [file pone.0281897.s001.pdf]

S1 Table. Search strategy. Recent queries in Databases on July 1 2022.

**Database**	**Search strategy:**	
**Web Of Science**	(TS=(Paraquat ）OR AB=(Methyl Viologen OR Gramoxone OR Paragreen A))AND (TS=(Creatinine) OR AB=(Krebiozen OR Creatinine Sulfate Salt OR kreatinin))	202
**Pubmed**	("Paraquat"[MeSH Terms] OR "Paraquat"[Title/Abstract] OR "methyl viologen"[Title/Abstract] OR "Gramoxone"[Title/Abstract] OR "paragreen a"[Title/Abstract]) AND ("Creatinine"[MeSH Terms] OR "Krebiozen"[Title/Abstract] OR (("creatinin"[All Fields] OR "Creatinine"[MeSH Terms] OR "Creatinine"[All Fields] OR "creatinines"[All Fields]) AND "sulfate salt"[Title/Abstract]) OR "Creatinine"[Title/Abstract])	121
**ScienceDirect**	(Paraquat OR Methyl Viologen OR Paragreen A OR Gramoxone) AND (Creatinine OR Krebiozen OR Creatinine Sulfate Salt OR kreatinin)	1019
**Embase**	(Paraquat OR Methyl Viologen OR Paragreen A OR Gramoxone) AND (Creatinine OR Krebiozen OR Creatinine Sulfate Salt OR kreatinin)	248
**Cochrane Library**	#1 paraquat<br>#2 (Methyl Viologen):ab,TI,kw OR (Gramoxone):ab,tI,kw OR (Paragreen A):ab,tI,kw<br>#3 #1 or #2<br>#4 Creatinine<br>#5 (kreatinin):ab,tI,kw OR (Krebiozen):ab,tI,kw OR (Creatinine Sulfate Salt):ab,tI,kw<br>#6 #4 or #5<br>#7 #3 and #6	11
**China National Knowledge Infrastructure**	(Topic: Paraquat (precise)) OR (Title, keywords and abstracts: Paraquat + Methyl viologen + Gramoxone + Paragrin A + Gramoxone (exact)) AND ((Topic: Creatinine (exact)) OR (Full text: Creatinine + Crebioson + Creatinine Sulfate Salt (exact)))	228
**China Online Journals**	(Topic: (paraquat) OR Title or keyword: (paraquat or methyl viologen OR gramoxone OR paragrin A OR gramoxone)) AND (Topic: (creatinine) OR Title or keyword: (creatinine OR crebioside OR creatinine sulfate salt))	189
**China Science and Technology**	title or keyword=paraquat or methyl viologen or gramoxone or paraglumine A or gramoxone AND title or keyword=creatinine OR crebioxin OR creatinine sulfate	11

**Journal Database**		
