# Supplementary material for: Serum creatinine in predicting mortality after paraquat poisoning: A systematic review and meta-analysis
Source: PLoS One. 2023 Feb 22;18(2):e0281897. doi: 10.1371/journal.pone.0281897 (PMC9946265; doi:10.1371/journal.pone.0281897)
Supplement: S2 Table — (PDF) [file pone.0281897.s002.pdf]

S2 Table. Quality assessment.

**PATIENTSELECTION**				**INDEXTEST(S)**			**REFERENCE STANDARD**			**FLOWAND TIMING**		
A	B	C		D	E		F	G		H	L	M
yes	yes	yes	low	yes	yes	low	yes	yes	low	yes	yes	yes
yes	yes	yes	low	yes	unclear	low	yes	yes	low	yes	yes	yes
yes	yes	yes	low	yes	unclear	low	yes	yes	low	yes	yes	yes
yes	no	yes	low	yes	unclear	low	yes	yes	low	yes	yes	yes
yes	yes	yes	low	yes	unclear	low	yes	yes	low	yes	yes	yes
yes	no	yes	low	yes	unclear	low	yes	yes	low	yes	yes	yes
yes	no	yes	low	yes	unclear	low	yes	yes	low	yes	yes	yes
yes	no	yes	low	yes	unclear	low	yes	yes	low	yes	yes	yes
yes	no	yes	low	yes	unclear	low	yes	yes	low	yes	yes	yes
yes	yes	yes	low	yes	unclear	low	yes	yes	low	yes	yes	yes

*A: Was a consecutive or random sample of patients enrolled? B: Was a case-control design avoided? C: Did the study avoid inappropriate exclusions? D: Were the index test results interpreted without knowledge of the results of the reference standard? E: If a threshold was used, was it pre-specified? F: Is the reference standard likely to correctly classify the target condition? G: Were the reference standard results interpreted without knowledge of the results of the index test? H: Was there an appropriate interval between index test(s) and reference standard? L: Did all patients receive a reference standard? M: Were all patients included in the analysis?
