# Supplementary material for: Serum creatinine in predicting mortality after paraquat poisoning: A systematic review and meta-analysis
Source: PLoS One. 2023 Feb 22;18(2):e0281897. doi: 10.1371/journal.pone.0281897 (PMC9946265; doi:10.1371/journal.pone.0281897)
Supplement: S3 Table — (PDF) [file pone.0281897.s003.pdf]

S3 Table. Data extracted.

First_Author	Publication_Year	Male%	Region	Survivor	No-survivor	End Point (days))	Sensitivity	Specificity
Song	2020	45.60%	China	62	52	90	92.31%	62.90%
Zhao	2020	43.50%	China	44	64	90	75%	88.60%
Wan	2017	52.50%	China	24	16	NA	94%	67.00%
Su	2020	52.60%	China	71	119	NA	75%	86.00%
chen	2011	35.60%	China	17	28	30	100%	88.20%
Xiao	2017	39.70%	China	33	25	90	62.50%	94.23%
Wang	2018	35.40%	China	102	60	NA	88.90%	58.80%
Wang	2011	56.10%	China	29	28	NA	89.30%	86.20%
Li	2014	48.80%	China	25	18	14	97.80%	66.70%
Kavousi	2017	66.30%	India	59	45	NA	90.90%	70.80%
