# Supplementary material for: Serum creatinine in predicting mortality after paraquat poisoning: A systematic review and meta-analysis
Source: PLoS One. 2023 Feb 22;18(2):e0281897. doi: 10.1371/journal.pone.0281897 (PMC9946265; doi:10.1371/journal.pone.0281897)
Supplement: S1 Fig — The diamond indicates the combined estimate from the included studies. (PDF) [file pone.0281897.s004.pdf]

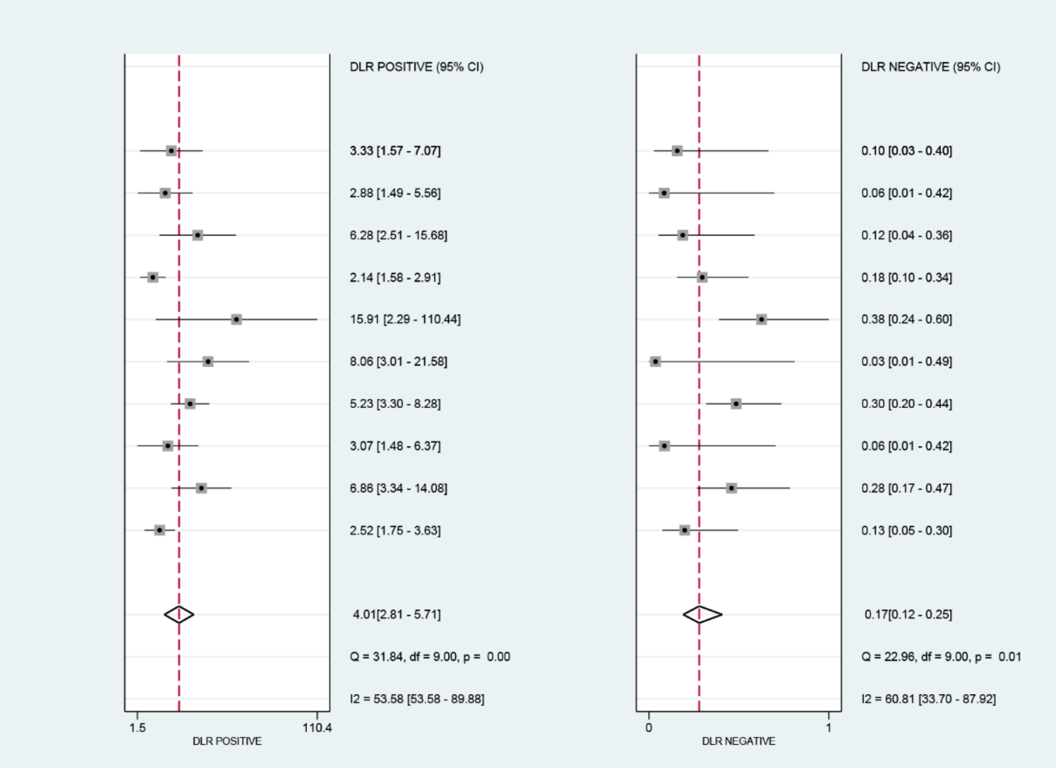

S1 Figure. Forest plots of positive likelihood ratio and negative likelihood ratio of Serum creatinine in predicting mortality after paraquat poisoning. The diamond indicates the combined estimate from the included studies.
