# Supplementary material for: Serum creatinine in predicting mortality after paraquat poisoning: A systematic review and meta-analysis
Source: PLoS One. 2023 Feb 22;18(2):e0281897. doi: 10.1371/journal.pone.0281897 (PMC9946265; doi:10.1371/journal.pone.0281897)
Supplement: S2 Fig — (A) Pre-test probability = 25%; (B) Pre-test probability = 50%; (C) Pre-test probability = 75%. Each Fagan plot contains a vertical axis on the left for the pre-test probability, an axis in the middle represents the likelihood ratio, and a vertical axis on the right represents the post-test probability. NLR: negative likelihood ratio, PLR: positive likelihood ratio. (PDF) [file pone.0281897.s005.pdf]

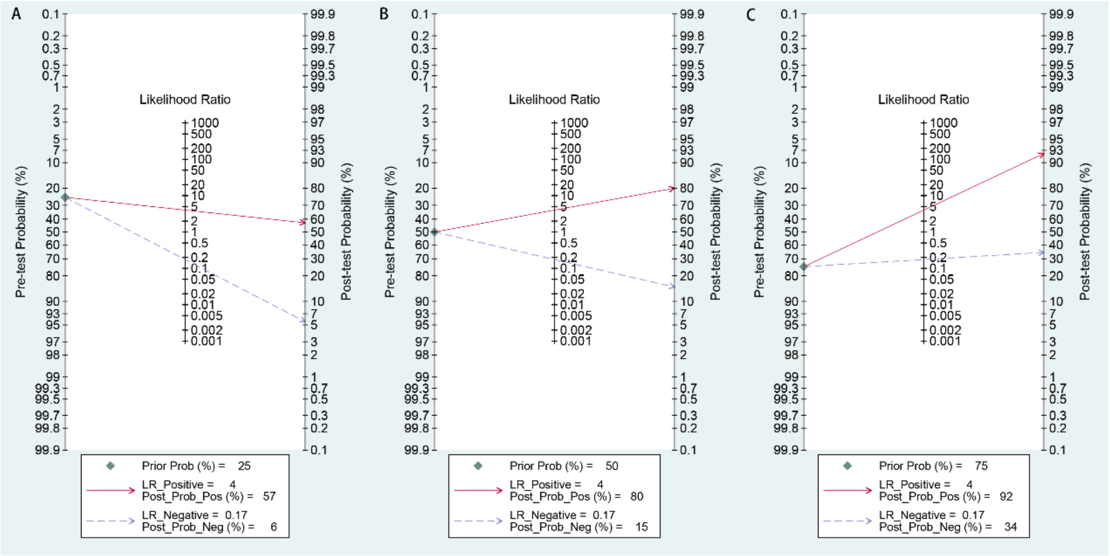

S2 Figure. Analysis of the Fagan plot to evaluate the clinical efficacy utility of serum creatinine in predicting mortality. (A) Pre-test probability=25%; (B) Pre-test probability=50%; (C) Pre-test probability=75%. Each Fagan plot contains a vertical axis on the left for the pre-test probability, an axis in the middle represents the likelihood ratio, and a vertical axis on the right represents the post-test probability. NLR: negative likelihood ratio, PLR: positive likelihood ratio.
